# Widely targeted metabolomics reveals the physiological and metabolic mechanisms of browning in tobacco leaves induced by starvation stress

**DOI:** 10.3389/fpls.2026.1752394

**Published:** 2026-03-26

**Authors:** Zeyu Zhao, Xueyan Jing, Guanhui Li, Qi Xu, Benbo Xu, Kesu Wei, Liping Chen, Bin Wei, Jiati Tang, Yuhang Zhao, Lingli Xie, Jianbing Qin, Shengjiang Wu

**Affiliations:** 1 Engineering Research Center of Ecology and Agricultural Use of Wetland, Ministry of Education/College of Life Sciences, Yangtze University, Jingzhou, China; 2 Upland Flue-Cured Tobacco Quality & Ecology Key Laboratory of China Tobacco, Guizhou Academy of Tobacco Science, Guiyang, China; 3 Suiyang Branch of Zunyi Company, Guizhou Provincial Tobacco Company, Zunyi, China; 4 Guizhou Provincial Tobacco Company, China National Tobacco Corporation, Guiyang, China

**Keywords:** browning mechanism, phenotypic physiology, starvation stress, tobacco, widely targeted metabolomics

## Abstract

Browning widely occurs during the processing of crops and their products, reducing both quality and economic value. However, the mechanisms underlying postharvest leaf browning under starvation-dominated conditions remain unclear. Here, compared with the standard curing practice (CK), lower tobacco leaves were subjected to a one-day dark treatment prior to curing to induce starvation-induced browning (SB), and were analyzed through integrated phenotypic characterization, enzyme activity assays, carbohydrate profiling, and widely targeted metabolomics during curing. The results showed that SB significantly intensified leaf browning and tissue shrinkage, accompanied by increased oxidative enzyme activities and malondialdehyde (MDA) content compared with CK during curing, as well as depletion of carbohydrate reserves, reflecting a pronounced metabolic imbalance. LC-MS/MS analysis identified a total of 812 metabolites in flue-cured tobacco leaves. Comparative analysis between CK and SB revealed 117 differentially accumulated metabolites (DAMs), including 73 up-regulated and 44 down-regulated metabolites. Notably, KEGG pathway analysis revealed extensive metabolic reprogramming, particularly involving in phenylpropanoid biosynthesis, flavone and flavonol biosynthesis pathways and phenylalanine, tyrosine and tryptophan biosynthesis pathways. It is worth emphasizing that SB treatment led to the accumulation of antioxidant-related metabolites (e.g., liquiritin and mangiferin) and the decrease in amino acids (e.g., phenylalanine, tryptophan and proline), highlighting the metabolic reprogramming underlying starvation-induced browning. Correlation analysis revealed significant correlations among metabolic changes, enzyme activities, browning severity, and water & structure traits. The results indicated that coordinated changes in enzymatic oxidation, substrate availability, and tissue water–structure properties are closely associated with postharvest leaf browning under starvation-dominated conditions, providing potential physiological and metabolic indicators for monitoring and mitigating browning in tobacco and other crops.

## 1 Introduction

As a major industrial crop with high economic value, tobacco (*Nicotiana tabacum* L.) is prone to browning during flue-curing and aging. It also serves as a model plant for studying postharvest physiology (Chen et al., 2022; Li et al., 2025b). Browning of tobacco leaves during curing often appears as brown to dark brown speckles, thus compromising their industrial value (Wang et al., 2024b). Compared with middle and upper leaves, lower leaves contain higher levels of organic acids and lower sugars contents. This composition tends to inhibit the Maillard reaction, but is conducive to the enhancement of polyphenol oxidase (PPO) activity. In addition, structural differences in epidermis tissues, cuticle thickness, and wax distribution may influence oxygen diffusion and enzyme–substrate interactions, potentially increasing susceptibility to early-curing browning (Hao et al., 2024).

Browning is usually caused by oxidation and polymerization of phenols into brown polymers based on studies in plants. This process may occur via two pathways: dependent on enzyme and independent on enzyme. The enzymatic pathway is primarily mediated by PPO and peroxidase (POD). The nonenzymatic pathway includes reactions such as the Maillard reaction, which is favored under conditions of high temperature, low pH, or in the presence of metal ion catalysts (Murata, 2021; Sui et al., 2023; Fraudentali et al., 2024). Beyond cosmetic defects, browning diminishes antioxidant capacity and alters metabolic and nutritional profiles, thereby complicating postharvest handling and processing of many agricultural products (Ismail et al., 2023; Wang et al., 2024a).

Among the factors associated with postharvest browning, carbon starvation has been widely implicated as a major contributing stress that predisposes tissues to enzymatic browning (Zhang et al., 2023a; Umeohia and Olapade, 2024).Tissue energy status tightly couples with browning development. Carbon shortage perturbs lipid and energy metabolism and induces starvation metabolism, characterized by enhanced autophagy, amino-acid reprogramming, and reactive oxygen species (ROS) accumulation (Goto-Yamada et al., 2019; Olmedilla and Sandalio, 2019; Wang et al., 2020). Energy deficiency inactivates membrane ion pumps and disrupts compartmentation, promoting contact between phenolic substrates and PPO (Luo et al., 2020). Previous studies indicated energy deficiency, membrane damage and browning are closely related (Gao et al., 2024; Zhao et al., 2024; Wang, 2025). Low-oxygen storage conditions affects carbon metabolism, reduces energy availability, and increases browning susceptibility in fruits (Boeckx et al., 2019). Collectively, energy dysregulation, ROS accumulation, and membrane impairment are frequently observed features associated with postharvest enzymatic browning (Wang et al., 2024a).

Despite these insights, the browning mechanisms underlying of tobacco leaves under starvation-dominated conditions during curing remain incompletely understood. In this study, lower tobacco leaves were pretreated with a one-day dark prior to curing to simulate starvation conditions. The tobacco leaves of CK and SB were analyzed with phenotypic characterization, enzyme activity assays, carbohydrate profiling, and widely targeted metabolomics. Specifically, this study characterized browning and structural changes in lower leaves. It also elucidated how carbon depletion stress drives the reprogramming of metabolic networks. Furthermore, we identified physiological and metabolic indicators that link energy status, membrane processes, and enzymatic browning. By providing insights into the coordinated relationships among secondary metabolism, membrane-associated processes, and browning development in an industrial crop during postharvest curing, this work advances our understanding of stress-related metabolic regulation. Furthermore, it offers practical strategies for optimizing curing management and quality control, thereby helping to mitigate value loss along the tobacco production chain.

## 2 Materials and methods

### 2.1 Plant material and sampling

The flue-cured tobacco cultivar Yunyan 87 (*Nicotiana tabacum* L.) was used in this study. Field trials were conducted at Heishi Town Science and Technology Park, Weining County, Bijie, Guizhou Province, China. Seedlings were transplanted in the 25th April at a spacing of 1.20 m × 0.50 m (row × plant) in soils of medium fertility. The experimental field (2.40 ha) was divided into six equal plots (0.40 ha each), managed according to local standard agronomic practices for high-quality flue-cured tobacco.

The fourth leaves from the bottom of tobacco plants were selected as experimental material. In each plot, 200 uniform and healthy plants were tagged prior to harvest. The experiment was conducted using two downdraft three-story bulk curing barns (8.00 m × 2.80 m × 3.50 m; length × width × height). At leaf maturity, three plots were randomly assigned to the control group and the remaining three plots were assigned to the dark pre-curing treatment group, resulting in three biological replicates per treatment.

**Figure 1:**
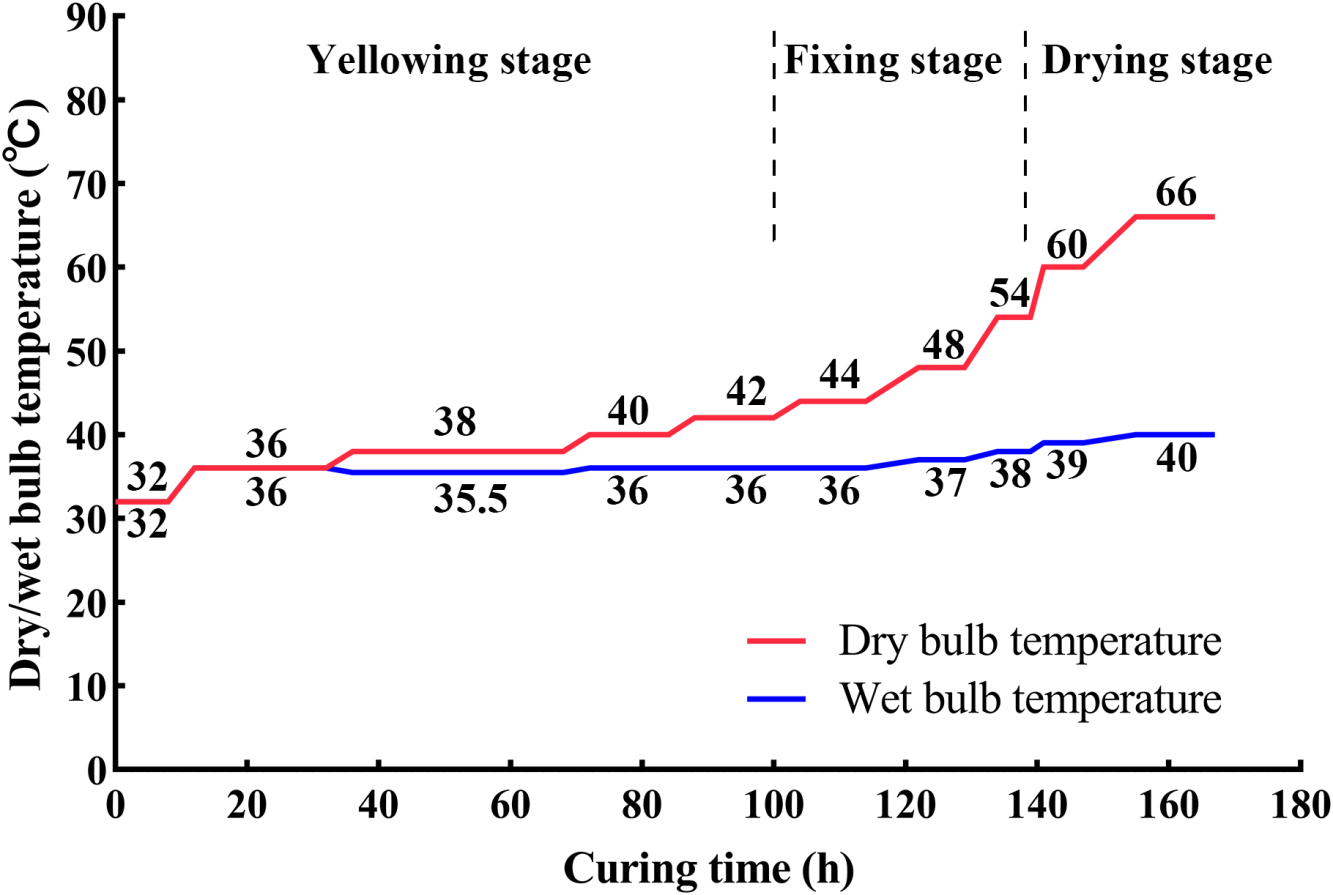
Dry-bulb and wet-bulb temperature profiles during flue-curing of tobacco leaves.

After harvest, leaves that were visibly damaged, showed signs of nutrient deficiency, or exhibited disease symptoms were discarded. Leaves from each plot were individually clamped using tobacco leaf clips, with three clips prepared per treatment group. Each curing barn was loaded with 300 clips of tobacco leaves. For CK, leaves were cured immediately following standard curing practice. For SB, harvested leaves were subjected to a one-day dark treatment at ambient temperature (22–25 °C) prior to flue-curing. CK samples were hung on the middle tier of one curing barn, while SB samples were placed at the same relative position in a separate barn. To minimize external airflow effects, one additional tobacco leaf clamp containing non-experimental leaves was placed between the sample clamps and the barn door as a buffer. Flue-curing was conducted according to the Guizhou “wilting–softening” three-stage protocol with ten key constant-temperature setpoints (**Figure 1**).

Samples were collected at four time points: fresh leaves before curing (Fresh), the end of the yellowing stage (42 °C), the end of the color-fixing stage (54 °C), and after curing (Cured). At each time point, samples with three biological replicates were collected. Each replicate was split into two portions: one portion was used for determination of leaf moisture content, color parameters, leaf shrinkage rate, and browning index, with leaves photographed for phenotypic analysis; the other portion was immediately frozen in liquid nitrogen and then stored at −80 °C for subsequent enzyme activity assays, physiological measurements, and widely targeted metabolomic analysis.

### 2.2 Measurements

#### 2.2.1 Leaf imaging and browning index

Representative leaves were sampled at the same four time points described above. Surface images were captured under standardized lighting and background using a Canon EOS 800D (Canon Inc., Tokyo, Japan). Following Upadhyay and Kumar (2021), browning areas were segmented in Adobe Photoshop 2021, and the browning index (BI) was calculated based on the ratio of browned-area pixels to total-leaf pixels. At least six leaves per group were analysed, and the mean BI was calculated.

#### 2.2.2 Color parameters and color difference

Leaf colour was measured using a portable CR-20 colorimeter (Konica Minolta, Tokyo, Japan) following Wu et al. (2020). Color data are reported as L* (lightness), a* (redness/greenness), and b* (yellowness/blueness) values, based on the CIELAB color space. L*, a*, and b* values were recorded for both adaxial and abaxial surfaces, and the total colour difference (ΔE*) was calculated as follows, with the subscripts 1 and 2 denoting the front and back surfaces, respectively.


$$\Delta E^{*}=\sqrt{{\left(L_{1}^{*}-L_{2}^{*}\right)}^{2}+{\left(a_{1}^{*}-a_{2}^{*}\right)}^{2}+{\left(b_{1}^{*}-b_{2}^{*}\right)}^{2}}$$

#### 2.2.3 Leaf shrinkage measurements

Leaf length (L) and leaf maximum width (W) were measured following the method of Zhu et al. (2014) and Wu et al. (2017). Longitudinal and lateral shrinkage rates (denoted as R_l_ and R_w_, respectively) were calculated according to Equation 1, with the previous and current time points denoted as 0 and 1, respectively.

(1) $$R_{l}\left(\%\right)=\left(1-\frac{L_{1}}{L_{0}}\right)\times 100\%,\;R_{w}\left(\%\right)=\left(1-\frac{W_{1}}{W_{0}}\right)\times 100\%$$

Fifteen biological replicates were included per time point. The approximate leaf area shrinkage, denoted as R_a_, was then computed from R_l_ and R_w_ as Equation 2:

(2) $$R_{a}\left(\%\right)=R_{l}+R_{w}-\frac{R_{l}\times R_{w}}{100}$$

#### 2.2.4 Enzyme activities and physiological indices

Leaf water content (LWC): Following Cai and Wu (2024), representative leaves were weighed immediately at each sampling point to determine fresh weight (FW). Samples were oven-dried at 80 °C in a forced-air oven for 6 h to constant mass and weighed for dry weight (DW). LWC was calculated as Equation 3:

(3) $$LWC\left(\%\right)=\left(1-\frac{DW}{FW}\right)\times 100\%$$

Enzyme activities and MDA content: The activities of PPO, phenylalanine ammonia-lyase (PAL), and POD, as well as the MDA content, were determined using commercial plant biochemical assay kits (Solarbio Life Sciences, Beijing, China) with the corresponding catalog numbers: PPO, cat. no. BC0195; PAL, cat. no. BC0215; POD, cat. no. BC0095; MDA, cat. no. BC0025. Measurements were performed on a Synergy H4 Hybrid microplate reader (BioTek Instruments) strictly following the manufacturer’s instructions. Readings were taken at 420 nm (PPO/POD), 290 nm (PAL), and 532 nm with 600 nm (MDA). Results were expressed on a fresh-weight basis (units per kit specification), with three biological replicates.

#### 2.2.5 Carbon-source compounds

The contents of soluble sugars, reducing sugars, and starch were determined as previously described by Chow and Landhäusser (2004), with minor modifications. Briefly, 0.20 g of post-cured tobacco leaf powder (dry basis) was extracted with 80% ethanol. Soluble sugars in the supernatant were quantified using the anthrone method (620 nm), whereas reducing sugars were determined using the 3,5-dinitrosalicylic acid (DNS) method (540 nm), with glucose as the standard. The ethanol-insoluble residue was sequentially hydrolyzed with α-amylase and amyloglucosidase (glucoamylase); released glucose was quantified by the anthrone method and converted to starch (glucose × 0.90). Absorbance was read on a microplate reader, and results were expressed on a dry-weight basis with three biological replicates.

#### 2.2.6 Widely targeted metabolomics

Extraction: Metabolites were extracted as previously described by Willency et al. (2024), with minor modifications. Briefly, frozen tobacco leaf tissue (150 mg) was extracted with 1.0 mL 70% methanol (tissue-to-solvent 1:6.7, w/v). Homogenates were vortexed at 4 °C every 10 min (three times) and kept overnight at 4 °C. The next day, extracts were centrifuged (13,000 ×g, 10 min, 4 °C), and supernatants were filtered through 0.22 µm PTFE organic-compatible membranes prior to LC–MS analysis. Sample injection order was randomized across groups, and a pooled QC sample was prepared by mixing equal aliquots of all samples and injected periodically.

LC–MS/MS: Metabolites were separated on a Waters HSS T3 column (2.1 × 100 mm, 1.8 µm; Waters, Milford, MA, USA) as in Wu et al. (2010). Mobile phases: 0.1% formic acid–water (A) and 0.1% formic acid–acetonitrile (B). Gradient: 0–2.00 min, 5% B; 2.00–22.00 min, linear to 95% B; 22.00–27.00 min, hold 95% B; 27.10–30.00 min, re-equilibrate 5% B. Column 40 °C; flow 0.30 mL min^-^¹; autosampler 4 °C; injection volume 2 µL. MS: QTRAP 6500 Plus (AB Sciex, Framingham, MA, USA) with ESI; source 450 °C; ion-spray +5500 V/−4500 V; GS1 = 40 psi, GS2 = 40 psi, CUR = 20 psi. Data were acquired in MRM with separate positive/negative runs; for each target, precursor/product ions, CE, DP, and retention time were recorded.

Data processing & statistics: Raw files were processed in Skyline v21.1.0.146 and matched to the BGI-Widetarget-Library to obtain identities and quantitative peak areas. In Meta X, features with >50% missing in QC or >80% missing in study samples were removed; remaining missing values were imputed by KNN; data were normalized by PQN; batch effects were corrected by QC-RLSC; features with QC CV > 30% were discarded. A QC was injected after every three samples and stability was assessed by PCA clustering. Multivariate analyses (PCA, OPLS-DA) were performed in SIMCA 14.1 with 200-time permutation testing. Differential metabolites were defined by fold change ≥ 2.0 or ≤ 0.5 with two-sided t-test, *p<* 0.05, and visualized by volcano plots. Pathway annotation used KEGG (https://www.kegg.jp/); bubble and pathway plots were generated in MetaboAnalyst 5.0. Metabolites with low signal intensity, marginal fold changes, or insufficient MS/MS information were retained for statistical completeness but were interpreted with caution and excluded from mechanistic inference.

### 2.3 Data analysis

Primary data organization and preliminary calculations were performed in Microsoft Excel 2021 (Microsoft Corp., Redmond, WA, USA). Statistical analyses were conducted in R (R Core Team, Vienna, Austria) using standard statistical procedures. Figures were prepared using GraphPad Prism 9.5 (GraphPad Software, San Diego, CA, USA) and Adobe Illustrator 2024 (Adobe Inc., San Jose, CA, USA). Unless otherwise specified, data are presented as mean ± SE of biological replicates. Differences among treatments were evaluated by one-way ANOVA, followed by the least significant difference (LSD) *post hoc* test when ANOVA indicated overall significance level of *p*< 0.05. Mantel analysis and the corresponding correlation heatmaps were generated using the online bioinformatics platform (https://www.bioinformatics.com.cn/) with permutation-based testing and default distance metrics to assess associations between metabolite profiles and physicochemical indices.

## 3 Results

### 3.1 Browning phenotypes and associated water–structure changes during curing

#### 3.1.1 Visual browning phenotypes

**Figure 2:**
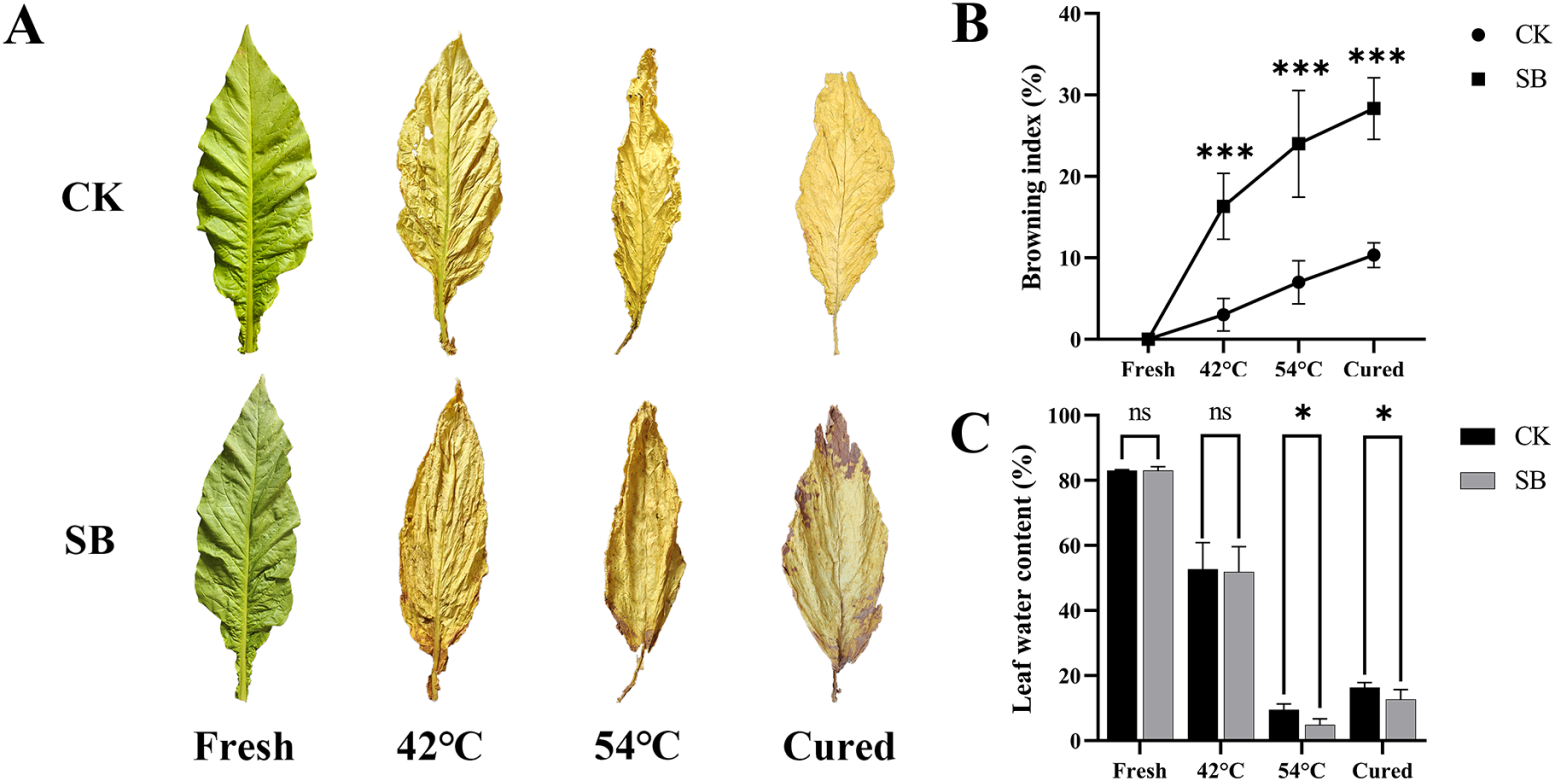
Phenotypic responses to pre-curing starvation: browning progression **(BI)** and leaf water content during curing. **(A)** Representative phenotypes across curing stages. **(B)** Browning index (BI) of SB and CK leaves at each stage. **(C)** Leaf water content (LWC). Error bars, mean ± SE; n = 4 biological replicates. Statistical annotations: ns, non-significant; *, and *** denote differences vs. CK at *p*< 0.05, and *p*< 0.001, respectively.

As shown in **Figure 2A**, leaves subjected to the starvation treatment developed numerous dark speckles at the leaf tip or base, coinciding with the end of the yellowing stage (42 °C). As curing temperature increased, speckles spread from the leaf margin toward the midrib. By the end of the color-fixing stage (54 °C), the speckles had expanded to cover most of the adaxial surface and deepened into a darker brown; they were also visible on the abaxial side. In contrast, CK displayed no obvious browning throughout the entire curing process.

Consistent with the visual symptoms, the browning index (BI) increased with temperature in both groups but was significantly higher in SB than in CK (**Figure 2B**). At 42 °C, 54 °C, and after curing, the BI of SB was significantly higher than that of CK (*p*< 0.001), indicating that pre-curing starvation was associated with an earlier onset and greater accumulation of browning during curing.

#### 3.1.2 Color parameters

To characterize color development and browning, L*, a*, b*, and C* were measured on both adaxial and abaxial surfaces across curing stages, with the inter-surface color difference ΔE* calculated (**Table 1**). At 42 °C, SB exhibited significantly lower values for L* (−8.87%), b* (−16.11%), and C* (−14.43%) compared to CK. At 54 °C, a* in SB increased by 29.99% (*p*< 0.01), while b* and C* significantly decreased (both *p*< 0.01), indicating an accelerated color shift from yellow to red–brown hues. Despite no further decrease in L* at 54 °C relative to 42 °C, ΔE* declined markedly, suggesting a reduction in front-back contrast due to more uniform browning across the lamina. Following starvation (SB), cured leaves displayed significantly reduced L* (−5.14%), b* (−6.96%), and C* (−6.12%) values compared to CK (*p*< 0.05), along with a 32.85% lower ΔE* (*p*< 0.001). These alterations demonstrate that pre-curing starvation led to distinct shifts in color development and browning.

**Table 1:** Effects of starvation treatment on leaf color parameters. Statistics annotations: ns, not significant; *, **, *** denote *p*< 0.05, *p*< 0.01, and *p*< 0.001, respectively.

Sampling	Treatment	L*	a*	b*	C*	ΔE*
Fresh	\	47.78 ± 1.74	-10.99 ± 0.15	34.78 ± 1.67	36.48 ± 1.55	8.23 ± 0.45
42 °C	CK	69.43 ± 0.73	7.99 ± 1.38	52.57 ± 3.97	53.18 ± 4.12	10.48 ± 2.00
	SB	62.13 ± 4.71*	10.93 ± 2.96	45.68 ± 2.63*	47.07 ± 1.95*	8.08 ± 2.28
54 °C	CK	67.64 ± 1.24	9.27 ± 1.42	53.04 ± 1.46	53.86 ± 1.47	10.73 ± 1.16
	SB	66.27 ± 1.75	12.05 ± 1.03**	48.88 ± 1.42**	50.34 ± 1.49**	9.02 ± 1.80
Cured	CK	68.84 ± 0.70	12.20 ± 0.71	55.77 ± 2.86	57.09 ± 2.92	13.09 ± 1.59
	SB	65.30 ± 1.28**	13.39 ± 0.97	51.88 ± 1.85*	53.59 ± 1.72*	8.79 ± 1.23**

#### 3.1.3 Water status and leaf shrinkage

LWC showed no significant difference between the SB and CK at 42 °C, but SB leaves exhibited lower LWC at 54 °C and after curing (*p*< 0.05; **Figure 2C**). The accelerated water loss showed a tight temporal coupling with enhanced browning severity in SB tobacco leaves during curing, implying that the altered water status may be closely associated with browning progression.

**Figure 3:**
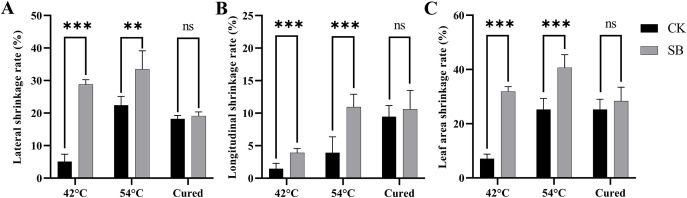
Effects of starvation stress on shrinkage rate of tobacco leaves. **(A)** Longitudinal shrinkage rate; **(B)** lateral shrinkage rate; **(C)** leaf-area shrinkage rate. Error bars, mean ± SE; n = 4. Statistical annotations: ns, not significant; **, *** indicate *p*< 0.01, and *p*< 0.001 vs. CK at the same stage, respectively.

At 42 °C, the lateral, longitudinal, and leaf area shrinkage rates of tobacco leaves in SB were all significantly higher than those of CK at 42 °C (*p*< 0.001; **Figure 3**). At 54 °C, SB still showed greater longitudinal and area shrinkage (*p*< 0.001) and significantly higher lateral shrinkage (*p*< 0.01). These patterns suggest that pre-curing starvation depleted internal reserves, leading to accelerated leaf tissue contraction during the early and middle curing phases.

### 3.2 Physiological responses to starvation treatment during curing

#### 3.2.1 Antioxidant enzyme activities and MDA accumulation

**Figure 4:**
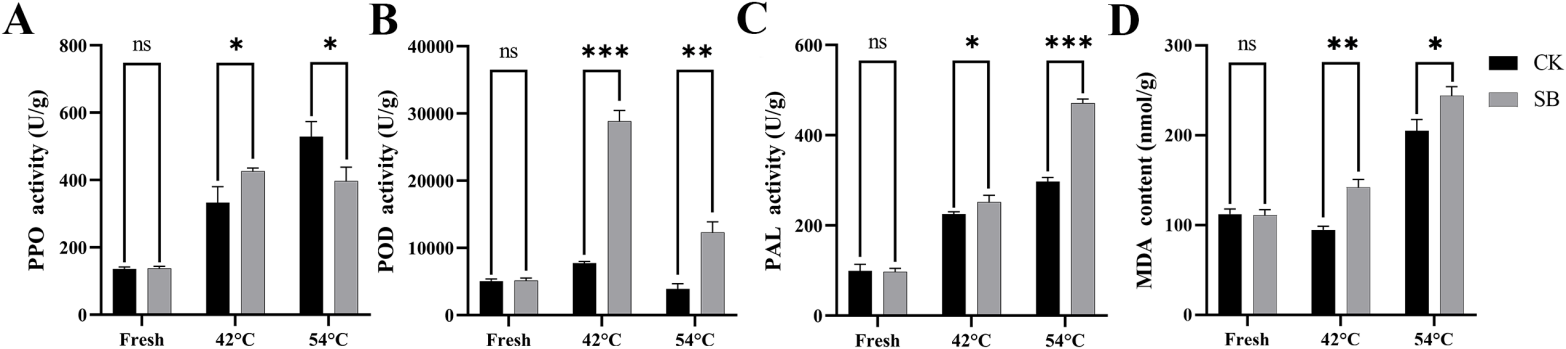
Effects of starvation treatment on antioxidant enzyme activities and MDA content. **(A)** PAL activity; **(B)** POD activity; **(C)** PPO activity; **(D)** MDA content. Data are mean ± SE (n = 3). Statistical annotations: ns, not significant; *, **, *** indicate *p*< 0.05, *p*< 0.01, and *p*< 0.001 vs. CK at the same stage, respectively.

As shown in **Figure 4**, at 42 °C, the activities of PPO (*p*< 0.05), POD (*p*< 0.001), and PAL (*p*< 0.05) were significantly higher in SB tobacco leaves compared to CK. At 54 °C, PPO activity in SB tobacco leaves was significantly lower than in CK (*p*< 0.05). In particular, PPO exhibited a biphasic response, with higher activity in SB at 42 °C but lower activity at 54 °C relative to CK. By contrast, the activities of POD (*p*< 0.01) and PAL (*p*< 0.001) remained elevated relative to CK. MDA content showed a progressive increase in both groups throughout curing. However, SB levels diverged from CK, becoming significantly higher at both 42 °C and 54 °C. These results indicate that SB treatment increased membrane lipid peroxidation and triggered stronger antioxidant and defense responses.

#### 3.2.2 Carbon-source compounds

**Figure 5:**
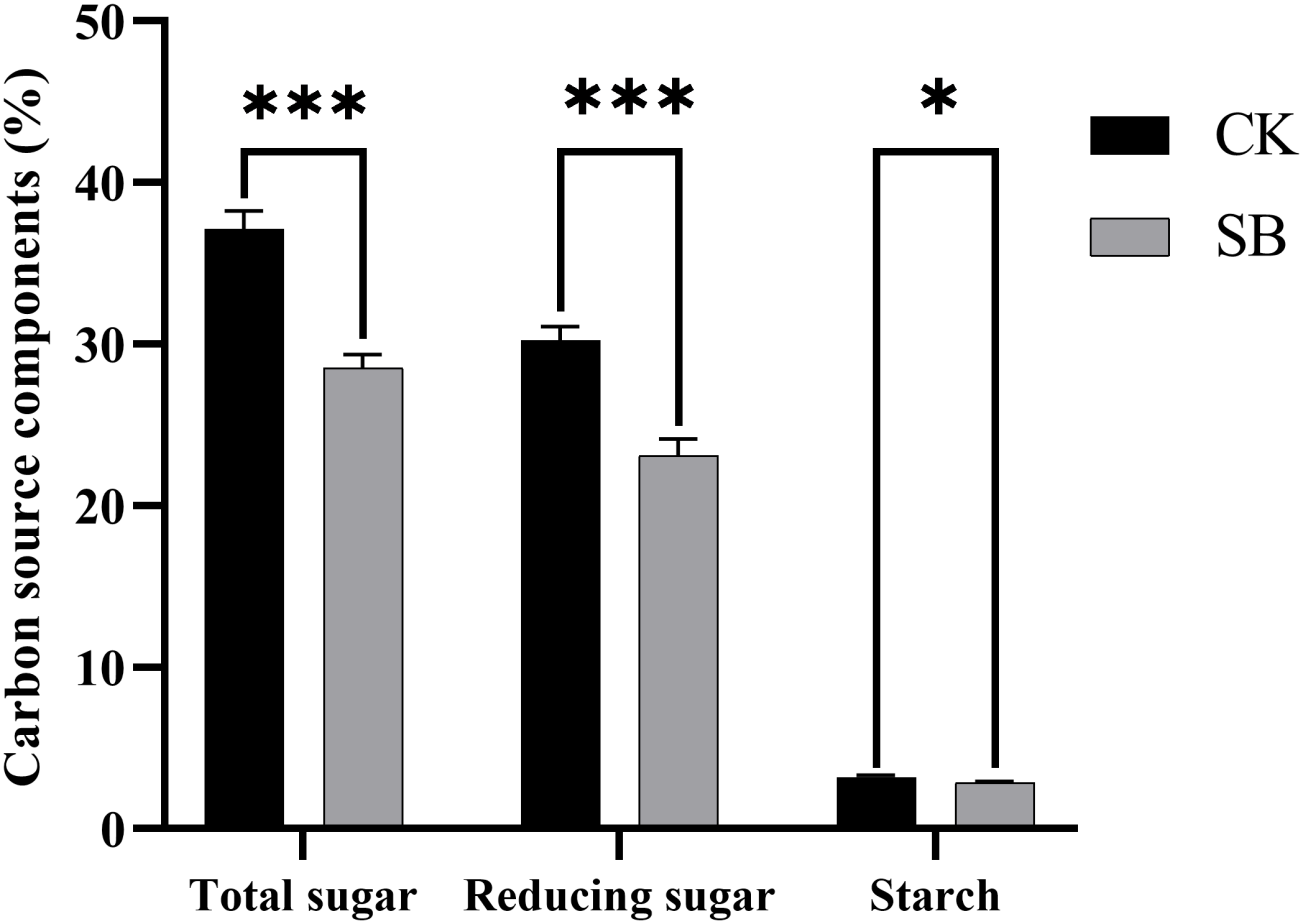
Effects of starvation treatment on carbon-source contents (total sugars, reducing sugars, starch) in cured leaves. Bars show mean ± SE (n = 3). ns, not significant; * and *** denote significant differences vs. CK at *p*< 0.05 and *p*< 0.001, respectively.

A significant depletion of carbon reserves was observed in SB tobacco leaves following curing, relative to CK (**Figure 5**). This was characterized by marked reductions in both total sugars (−23.22%, *p*< 0.001) and reducing sugars (−23.60%, *p*< 0.001), coupled with a significant decrease in starch content (−10.34%, *p*< 0.05). These results demonstrated substantial depletion of carbon reserves under pre-curing starvation, indicating a reduced availability of soluble carbohydrates during curing.

### 3.3 Metabolic responses to starvation treatment during curing

**Figure 6:**
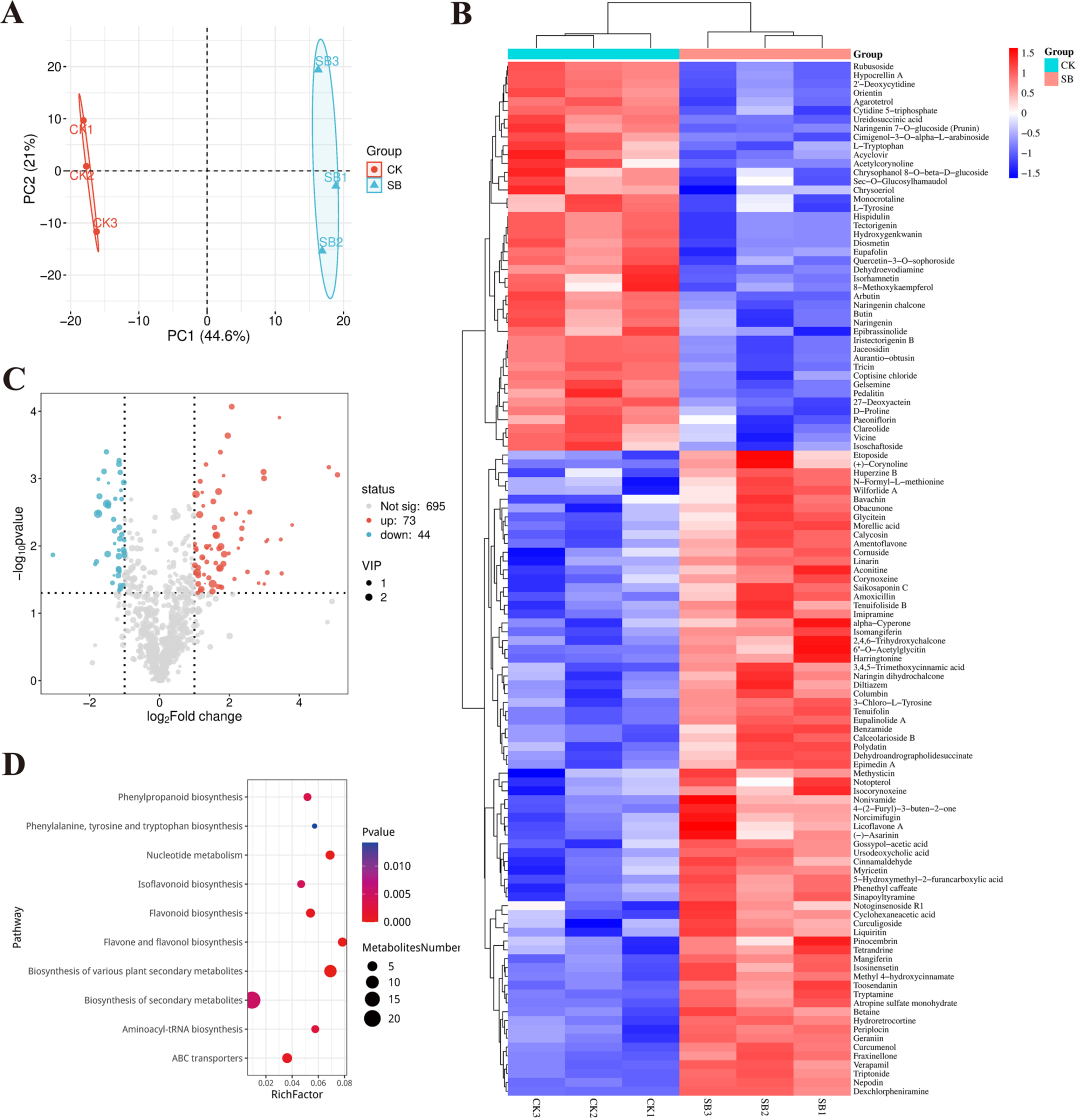
Multivariate analysis and characteristics of differential metabolites in cured tobacco leaves under starvation-induced browning. **(A)** PCA score plot. **(B)** Heatmap of significantly different metabolites (DAMs). **(C)** Volcano plot showing up- and down-regulated DAMs (criteria: *p*< 0.05; FC ≥ 2.0 or ≤ 0.5). **(D)** KEGG pathway enrichment of DAMs (bubble plot).

The PCA score plot (**Figure 6A**) showed clear separation between SB and CK, suggesting that the SB treatment had a significant impact on the metabolome. A total of 812 metabolites were identified based on positive and negative ion modes. Among them, 117 metabolites showed significant differences in accumulation between SB and CK (*p*< 0.05; FC ≥ 2.0 or ≤ 0.5), including 73 up-regulated and 44 down-regulated metabolites. The clustered heatmap (**Figure 6B**) and volcano plot (**Figure 6C**) visualize these distribution patterns. Several phenolic- and flavonoid-related compounds exhibited pronounced accumulation in SB, with mangiferin showing the highest fold change (FC = 34.16). Other highly responsive metabolites included tryptamine, linarin, liquiritin, and eupalinolide A, representing multiple classes of secondary metabolites. In contrast, a subset of metabolites showed significant decreases in SB relative to CK, including cytidine 5′-triphosphate and coptisine chloride. A small number of down-regulated features were annotated as non-canonical compounds; these features were characterized by low signal intensity and were therefore interpreted with caution and not emphasized in subsequent analyses.

KEGG pathway enrichment analysis of the DAMs highlighted significant enrichment in phenylpropanoid and flavonoid biosynthesis pathways (**Figure 6D**). This enrichment pattern was driven by DAMs primarily comprising phenolic and flavonoid compounds. The broad category of biosynthesis of secondary metabolites contained the largest number of DAMs, reflecting coordinated changes across multiple secondary metabolic branches. Additional enrichment was observed for flavone and flavonol biosynthesis, as well as isoflavonoid biosynthesis. Furthermore, enrichment of phenylalanine, tyrosine and tryptophan biosynthesis pathways indicated altered amino acid-associated metabolic profiles under starvation conditions. These results demonstrate that SB treatment is associated with extensive remodeling of secondary metabolite composition, particularly within phenylpropanoid and flavonoid biosynthetic pathways.

### 3.4 Integrated metabolic network analysis reveals associations with browning development

**Figure 7:**
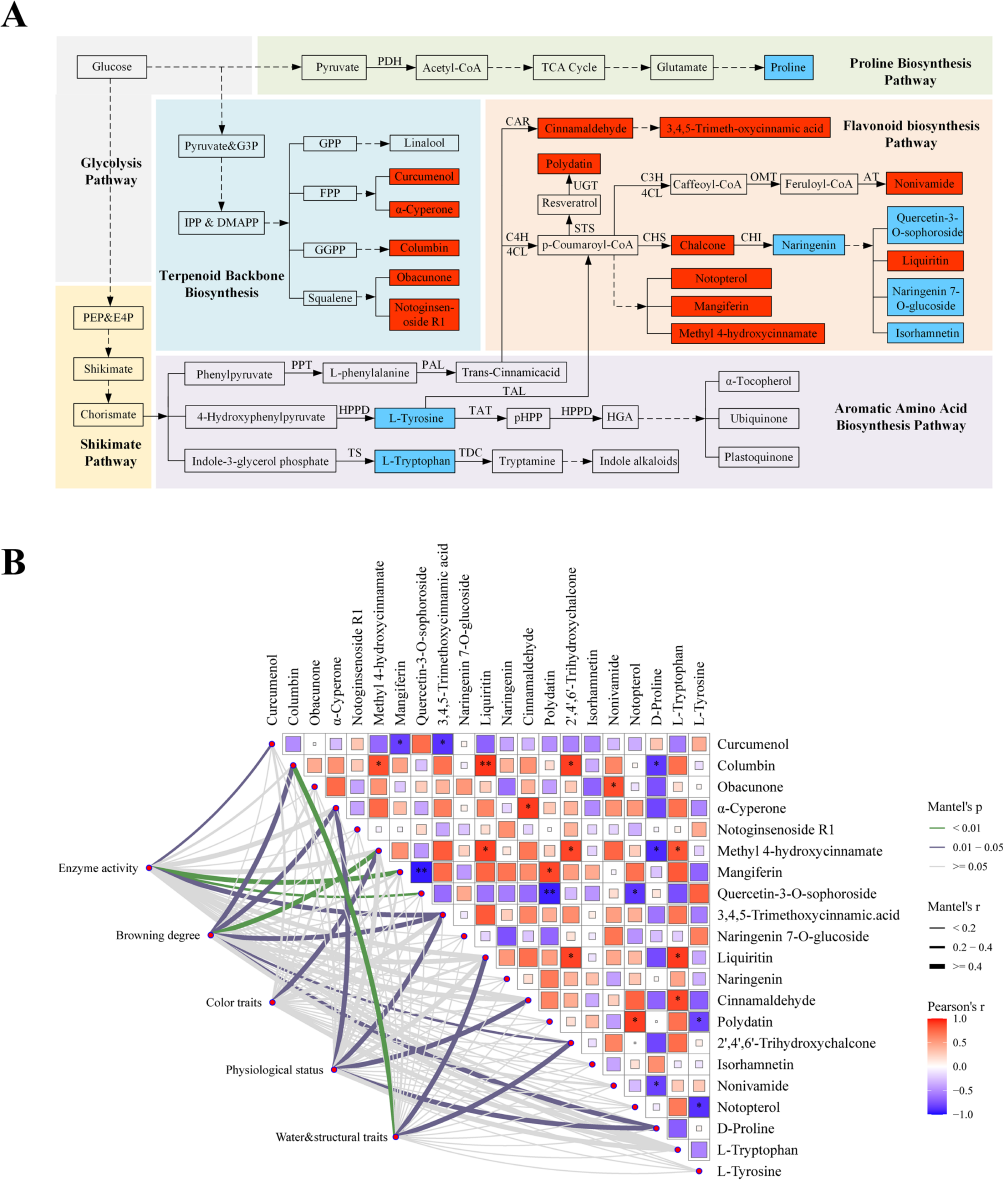
Personalized metabolic network and Mantel correlations under starvation-induced browning. **(A)** KEGG-based schematic highlighting glycolysis, shikimate–aromatic amino acid biosynthesis, phenylpropanoid, flavonoid, and terpenoid pathways; key intermediates include phosphoenolpyruvate (PEP), erythrose-4-phosphate (E4P), glyceraldehyde-3-phosphate (G3P), isopentenyl diphosphate (IPP), dimethylallyl diphosphate (DMAPP), geranyl diphosphate (GPP), farnesyl diphosphate (FPP), and geranylgeranyl diphosphate (GGPP). **(B)** Mantel correlation heatmap between metabolite composition and physicochemical/physiological traits; color scale denotes Mantel *r* (red, positive; blue, negative); * and ** indicate *p*< 0.05 and *p*< 0.01, respectively.

To examine coordinated metabolic alterations associated with browning under starvation conditions, DAMs were mapped onto KEGG-classified metabolic pathways (**Figure 7A**), including glycolysis, the shikimate pathway, aromatic amino acid biosynthesis, phenylpropanoid metabolism, and flavonoid and terpenoid biosynthesis. The SB treatment was associated with broad changes across these interconnected metabolic modules. Several metabolites in central carbon metabolism, such as glycolytic intermediates and D-proline, exhibited reduced relative abundance under starvation (SB) compared to the control (CK). Notably, pyruvate levels were also lower, which coincided with decreased concentrations of the aromatic amino acids phenylalanine, tyrosine, and tryptophan. These coordinated shifts indicate a starvation-induced reprogramming of carbon flux, culminating in altered metabolite availability at the shikimate–aromatic amino acid node. This upstream restriction of phenylalanine flux, in particular, provides a mechanistic basis for the subsequent remodeling of downstream phenolic metabolism.

Within the phenylpropanoid network, this altered precursor availability led to branch-specific outcomes: while upstream intermediates related to p-coumaroyl-CoA formation (e.g., methyl 4-hydroxycinnamate, cinnamaldehyde) were more abundant, the accumulation of multiple downstream flavonoids and chalcones (e.g., tricin, hispidulin, isorhamnetin, naringenin, and 2,4,6-trihydroxychalcone) was reduced. In contrast, phenylpropanoid-derived phenolic glycosides (e.g., polydatin, liquiritin) and selected oxidized derivatives were significantly enriched in SB. This branch-specific reprogramming highlights how starvation stress differentially regulates competing pathways within the phenylpropanoid network. Beyond phenylpropanoids, starvation induced coordinated changes across other classes of secondary metabolites. The relative abundance of several terpenoid-related compounds (e.g., α-cyperone, columbin), coumarins (e.g., notopterol), and polyketide-type metabolites (e.g., mangiferin, methysticin) was elevated in SB leaves. This broad activation suggests a systemic stress response that redirects metabolic resources toward diverse specialized metabolite pathways.

To assess associations between metabolic variation and physiological traits, Mantel correlation analysis was performed between the metabolite abundance matrix and the physicochemical and physiological parameters (**Figure 7B**). Most metabolites positively correlated with enzyme activities (PPO, POD, PAL), browning indices, and water & structural traits (Mantel r ≥ 0.4, *p*< 0.05), indicating that shifts in metabolite composition are tightly coupled to enzymatic activity and tissue water status. In contrast, associations between metabolites and color parameters (L*, a*, b*) and certain bulk physiological measures (e.g., sugars, MDA) were comparatively weaker (r< 0.2, *p* > 0.05). These results demonstrate strong correlations between starvation-induced browning and the concomitant shifts in secondary metabolite composition, enzymatic activity, and tissue physiological status.

Taken together, integrated network analysis reveals that starvation induces an extensive reorganization of the tobacco leaf metabolome, with pronounced shifts in phenylpropanoid, flavonoid, and terpenoid pathways. The coordinated changes in these metabolite pools, along with their close associations with key enzymatic activities and tissue physiological status, collectively demonstrate a strong linkage between the reprogramming of secondary metabolism and the progression of browning during flue-curing under starvation stress.

## 4 Discussion

Pre-curing starvation treatment reshaped leaf phenotype and metabolism, and accelerated enzymatic browning in tobacco leaves during flue-curing. A speckle-like browning, which initiated at the leaf tip or base and expanded along the margins toward the midrib, resembled high temperature stress associated spotting previously reported in tobacco leaves (Wang et al., 2024a), suggesting that starvation preconditioning sensitizes leaf tissues to subsequent high temperature stress during curing. These visual alterations were accompanied by increased dehydration and shrinkage during the yellowing and color-fixing stages, indicating that starvation compromises the structural integrity of leaf tissues prematurely. This increased shrinkage likely reflects an accelerated depletion of carbon and water reserves, thus decreasing osmotic potential and turgor and promoting dehydration-driven tissue collapse during curing. The similar findings have been reported in potato, litchi, and apple during postharvest period (Lurie and Mitcham, 2007; Zhang et al., 2016).

Building on the early physical destabilization and dehydration induced by pre-curing starvation, leaf browning progression during flue-curing was further driven by coordinated reprogramming of browning-related enzymes and oxidative status. At 42 °C and 54 °C, PAL activity was higher in SB than in CK, indicating activation of the phenylpropanoid pathway and enhanced secondary metabolism under oxidative pressure (Yadav et al., 2020; Polivanova and Cherednichenko, 2023). PAL exerts a dual influence on browning: on the one hand, supporting defense-related metabolic fluxes, and on the other, increasing the pool of phenolic substrates susceptible to enzymatic oxidation (Petersen et al., 2010; Li et al., 2023a). POD activity significantly increased at 42 °C and 54 °C during curing, consistent with its role in reactive oxygen species scavenging as well as phenolic oxidation and polymerization. Notably, PPO activity exhibited a biphasic response, increasing at 42 °C but declining at 54 °C. This pattern indicated that PPO did not act as a constant rate-limiting factor throughout flue-curing. Instead, PPO activation may initiate phenolic oxidation and quinone formation, whereas browning progression was likely governed by substrate availability, oxidative environment, and downstream polymerization processes at later stages. As curing proceeds, the depletion of oxidizable phenolics, accumulation of quinones, and partial thermal inactivation of PPO may work together to decouple PPO activity from visible browning intensity. Similar stage-dependent PPO behavior has been reported in thermally processed plant tissues, where browning persists despite reduced PPO activity once oxidative cascades are established (Lurie and Mitcham, 2007; Zhang et al., 2016). Prior work similarly highlighted the coordination between phenylpropanoid flux and reactive oxygen species metabolism in regulating browning and stress resistance (Zhang et al., 2025), and it shows that oxidative stress can shift the balance between defense enzymes (PAL, POD, CAT) and pro-browning enzymes (PPO, LOX) to determine browning severity (Li et al., 2023b). In agreement, MDA was higher in SB at 42 °C and 54 °C, indicating intensified membrane lipid peroxidation and oxidative stress (Kandlbinder et al., 2004). Together, these enzymatic and oxidative indicators suggested that pre-curing starvation enhances antioxidant and defense programs while simultaneously facilitating enzymatic browning through increased phenolic availability and altered membrane status. This highlights an intrinsic trade-off between stress defense activation and dark or black pigment formation in tobacco leaves during curing.

On the basis of the observed enzyme kinetics and oxidation reactions, the availability of carbon sources became the critical limiting factor for browning development under starvation conditions. Starvation significantly decreased the contents of total sugars, reducing sugars, and starch in cured leaves, indicating pronounced carbon depletion and a weakened capacity to maintain energy metabolism and cellular homeostasis under stress. This pattern is consistent aligned with a broader phenomenon of postharvest fruit browning in which carbon and energy imbalances are strongly associated with lipid oxidative browning. For instance, Zhang et al. (2023b) showed that maintaining respiratory efficiency in strawberry preserved soluble sugars and delayed browning, while Qi et al. (2022) reported that disrupted starch–sugar balance contributed to peel browning in pomegranate. In apple, Li et al. (2025a) demonstrated that alleviation of browning was associated with stabilization of energy metabolism and antioxidant capacity, accompanied by reduced PPO activity. Similarly, Wang et al. (2023) found that coordinated down-regulation of glycolysis and gluconeogenesis, altered starch and sucrose metabolism, and impaired ROS scavenging jointly promoted flesh browning in cold-stored ‘Fuji’ fruit. Taken together, these studies and our results indicate that carbon starvation disrupts cellular energy charge and membrane integrity, leading to the oxidation of phenolic substrates and enzymatic browning. In flue-cured tobacco curing, premature carbohydrate depletion predisposes leaf tissue to oxidative damage and enhances enzyme-substrate interaction during dehydration, leading to browning. This mechanism offers a rationale for strategies designed to stabilize carbon and energy status before curing.

Consistent with the depletion of carbon reserves, metabolomic analysis revealed a pronounced reprogramming of primary and secondary metabolism under starvation. The overall, the metabolomic profile indicated a redistribution of metabolic resources toward stress-responsive secondary pathways. Specifically, metabolites associated with phenylpropanoid and related secondary metabolism were preferentially accumulated. This suggests an activation of defense-oriented metabolic programs that enhance antioxidant capacity and stress signaling under carbon-limited conditions (Xue et al., 2022). In contrast, the marked reduction in several amino acids, including proline, tyrosine, and tryptophan, suggests a restriction in the glycolysis-derived carbon flux directed toward amino acid biosynthesis. Amino acid metabolism plays a central role in stress signaling and responses to energy deficit (Heinemann and Hildebrandt, 2021). Under abiotic stress, proline typically accumulates, functioning as an osmoprotectant and antioxidant (Szabados and Savouré, 2010). However, its depletion in starved leaves likely results from a constrained biosynthetic capacity due to carbon limitation, coupled with its rapid consumption for redox buffering. These combined shifts represent an early metabolic signature of starvation-induced reprogramming, wherein the limited primary carbon is redirected. This redirection prioritizes the synthesis of stress-defense and browning-related secondary metabolites over the production of amino acids for growth.

In line with carbon depletion and constrained primary metabolism, pathway-level analysis revealed pronounced alterations at the shikimate–phenylpropanoid node. Aromatic amino acids (L-phenylalanine, L-tyrosine, L-tryptophan), synthesized from PEP and E4P via the shikimate pathway, serve as core precursors for phenylpropanoids, indole compounds, and alkaloids (Shende et al., 2024). Their coordinated decrease in SB suggests attenuation of the shikimate pathway, plausibly linked to limited availability of glycolysis-derived PEP under starvation. Consistent with this, p-coumaroyl-CoA represents a critical branch point that can either support flavonoid and volatile biosynthesis or be redirected to phenolic substrates, which are subsequently oxidized by PPO to generate quinones involved in enzymatic browning (Ma and Waterhouse, 2018). Indeed, flavonoid-associated metabolites were generally reduced, whereas antioxidant and defense related phenolic pools accumulated in SB, indicating a redistribution of phenylpropanoid metabolism under stress. This accumulation of phenolics aligns with their known role in antioxidant defense—a function documented for canonical phenolics including chalcones, stilbenes, and cinnamic-acid derivatives (Moazzen et al., 2022; Chen et al., 2023; Ghareghomi et al., 2023; Shahcheraghi et al., 2023; Cuchet et al., 2024), and is consistent with an adaptive metabolic response to starvation. In parallel, multiple terpenoids derived from the MEP and MVA pathways were also increased, covering sesquiterpenoid, diterpenoid, and triterpenoid classes. Given their biosynthetic reliance on acetyl-CoA and pyruvate precursors, this accumulation not only indicates stress-responsive activation of terpenoid metabolism, potentially contributing to antioxidant, antimicrobial, and membrane-protective roles (Chen et al., 2024), but also reflects a broader redistribution of primary carbon into secondary pathways under starvation.

**Figure 8:**
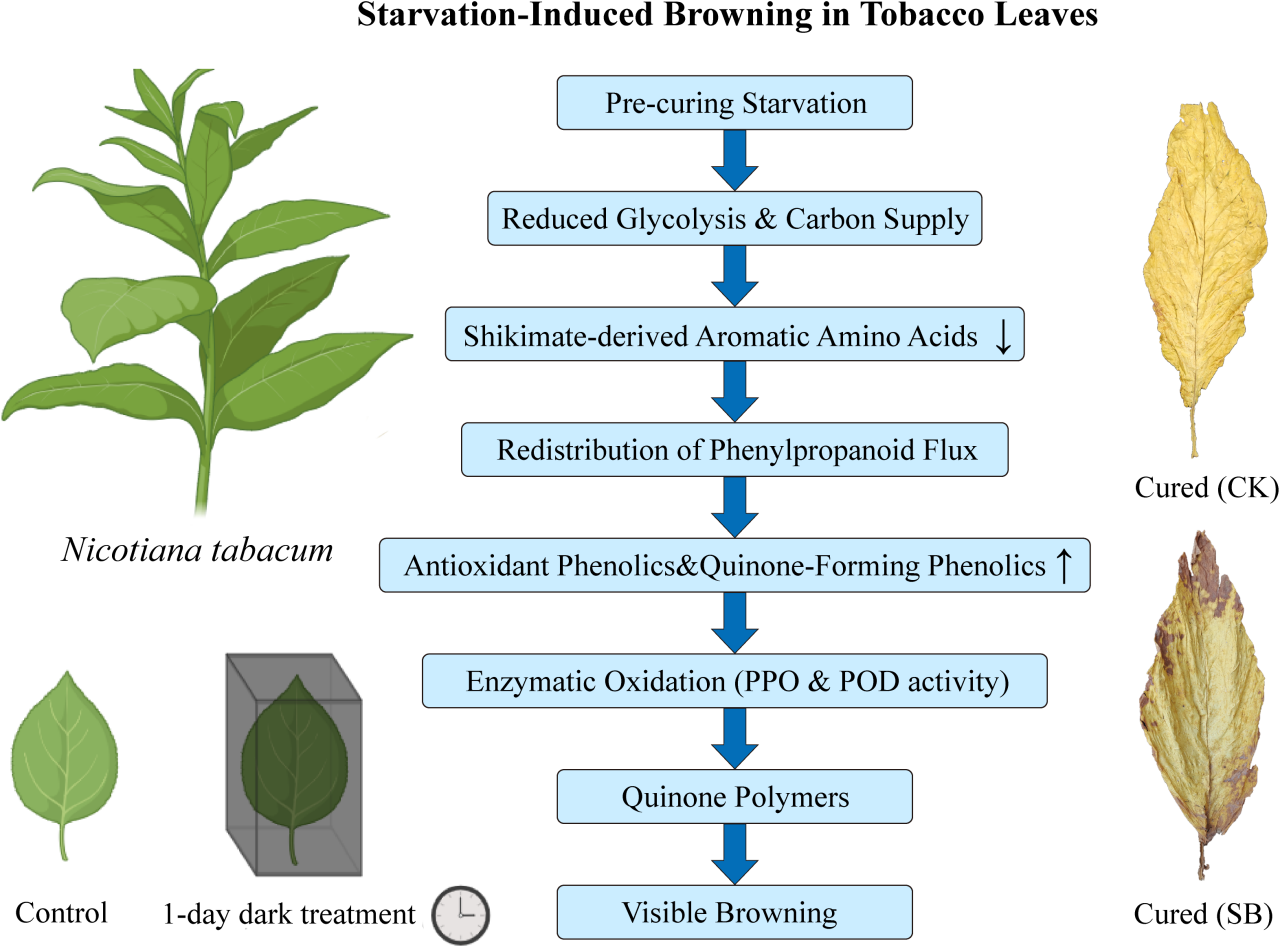
Conceptual mechanistic model of starvation-induced browning in flue-cured tobacco leaves.

Mantel correlation analysis indicated that metabolite differences were closely associated with physicochemical traits. Prominent phenolic and flavonoid shifts paralleled the enhanced PPO, POD, and PAL activities, while strong associations with water content and structural indices suggested that dehydration and tissue collapse may influence enzyme substrate proximity, thereby potentially contributing to enzymatic browning. These observations collectively support a conceptual sequence (**Figure 8**): carbon limitation under starvation suppresses glycolytic and shikimate pathways. This metabolic shift leads to a relative accumulation of antioxidant and quinone-forming phenolic pools, which, upon oxidation mediated by POD and PPO, may generate quinone polymers and melanin-like pigments, manifesting as visible browning (Fan, 2022; Singh et al., 2024). While this study provides an integrative view of starvation-induced browning in tobacco leaves, several limitations should be acknowledged. The pre-curing treatment represents a composite stress beyond carbon depletion, and direct measurements of cellular energy status and ROS were not performed. Metabolomics-based inferences, including flux shifts and the roles of partially annotated metabolites, are constrained by annotation confidence. Furthermore, only lower leaves at specific time points were analyzed, limiting generalization. Future work should directly assess energy and redox status to validate the proposed mechanistic framework and expand these findings across leaf positions and developmental stages.

## 5 Conclusions

To explore the browning mechanisms in postharvest tobacco leaves under starvation-dominated conditions, a comparative analysis was performed through integrated phenotypic characterization, enzyme activity assays, carbohydrate profiling, and widely targeted metabolomics, comparing a one-day dark treatment prior to curing with the standard curing practice. Comparative analyses revealed that pre-curing starvation reprogrammed tobacco leaf metabolism, reshaping leaf phenotypes and metabolic fluxes during flue-curing. Starvation was associated with constrained glycolysis, reduced shikimate-derived aromatic amino acids, and a redistribution of phenylpropanoid metabolism toward the biosynthesis of antioxidant phenolics and quinone-forming pathways. The concurrent decline in sugars and starch signaled a weakened energy supply, which could indirectly promote enzymatic browning by compromising cellular integrity and increasing substrate availability for oxidation. These findings provide mechanistic insights for potential strategies to mitigate browning and reduce quality loss, including maintaining carbohydrate status, regulating early dehydration, and targeting PPO/POD activity or phenylpropanoid metabolic flux.

## Data Availability

The raw data supporting the conclusions of this article will be made available by the authors, without undue reservation.
